# A preliminary clinical study of endoscopic minimally-invasive surgery in urethral stricture complicated with false passage

**DOI:** 10.1186/s40064-016-3137-x

**Published:** 2016-08-30

**Authors:** Wei Le, Weidong Zhou, Chao Li, Denglong Wu, Jinfu Zhang, Cuidong Bian

**Affiliations:** 1 Department of Urology, Tongji Hospital, Tongji University School of Medicine, 389 Xincun Road, Shanghai, 200065 China; 2 Department of Reproduction and Andrology, Tongji Hospital, Tongji University School of Medicine, 389 Xincun Road, Shanghai, 200065 China; 3 Department of Reproduction and Andrology, Tongren Hospital Affiliated to Shanghai Jiaotong University, Shanghai, 200050 China

**Keywords:** Endoscopic minimal invasive surgery, False passage, Urethral reconstruction, Urethral stricture

## Abstract

The aim of this study was to explore the clinical effect of endoscopic minimal invasive surgery on posterior urethral stricture with false passage. Twenty-one patients suffering from posterior urethral stricture with false passage were involved in the study. All the patients received pre-operative urethrography and flexible cystoscopy to make sure that the distance between the blind end of the proximal normal urethra and the distal urethra was <1 cm. Ten patients received open operation and eleven patients underwent endoscopic minimally-invasive surgery. All the patients in both groups had their catheters removed 4 weeks after operations, and improvements in urination and incontinence were observed. Urethrography was performed and urine flow rate was measured 1 month after catheter removal. In the open-operation group, nine patients showed unobstructed urinary tracts in the urethrography, and one, after his catheter removal, experienced dysuresia, which was improved after urethral dilatation. In the minimally-invasive operation group, nine patients showed patent urinary tracts in the urethrography, and two experienced post-operation dysuresia, of whom, open-operation treatment and urethral dilatation were performed respectively. In the minimally-invasive operation group, the average urine flow rate was significantly increased. Patients in both groups obtained obvious improvement in post-operation urinary incontinence, and there was no statistically significant difference between the two groups in urine flow rate and index for urinary incontinence. Endoscopic minimally-invasive operation had similar effects to open operation in treatment of posterior urethra stricture with <1 cm in length and false passage.

## Background

Pelvic fracture is the most common reason for posterior urethrostenosis (Hampson et al. 2014). Casual or multiple urethral dilations usually lead to urinary tract stricture complicated with false urethral passage (Veeratterapillay and Pickard 2012), which further leads to recurrent infection and incontinence. Urethrography and flexible cystoscope are helpful for definite diagnosis (Hosseini et al. 2006).

Conventional open operation is the most common method for treatment of posterior urethral stricture complicated with false passage (Fu et al. 2011), but it is also always accompanied with more short-term or long-term complications, such as big operative wound and postoperative sexual function loss, etc. (Blaschko et al. 2013; Carlton et al. 2008). In this study, patients with stenotic segments of <1 cm in length were randomly divided into two groups. Endoscopic minimally-invasive operation or open operation was performed, and treatment outcomes were compared.

## Methods

A total of 21 patients suffering from urethral stricture with false passage were admitted in our hospital from 2002 to 2014, and the mean age was 36.25 years old (22–59 years). All of them developed posterior urethral stricture because of pelvic fracture, and had a history of urethral dilatation (1–6 times).

**Fig. 1:**
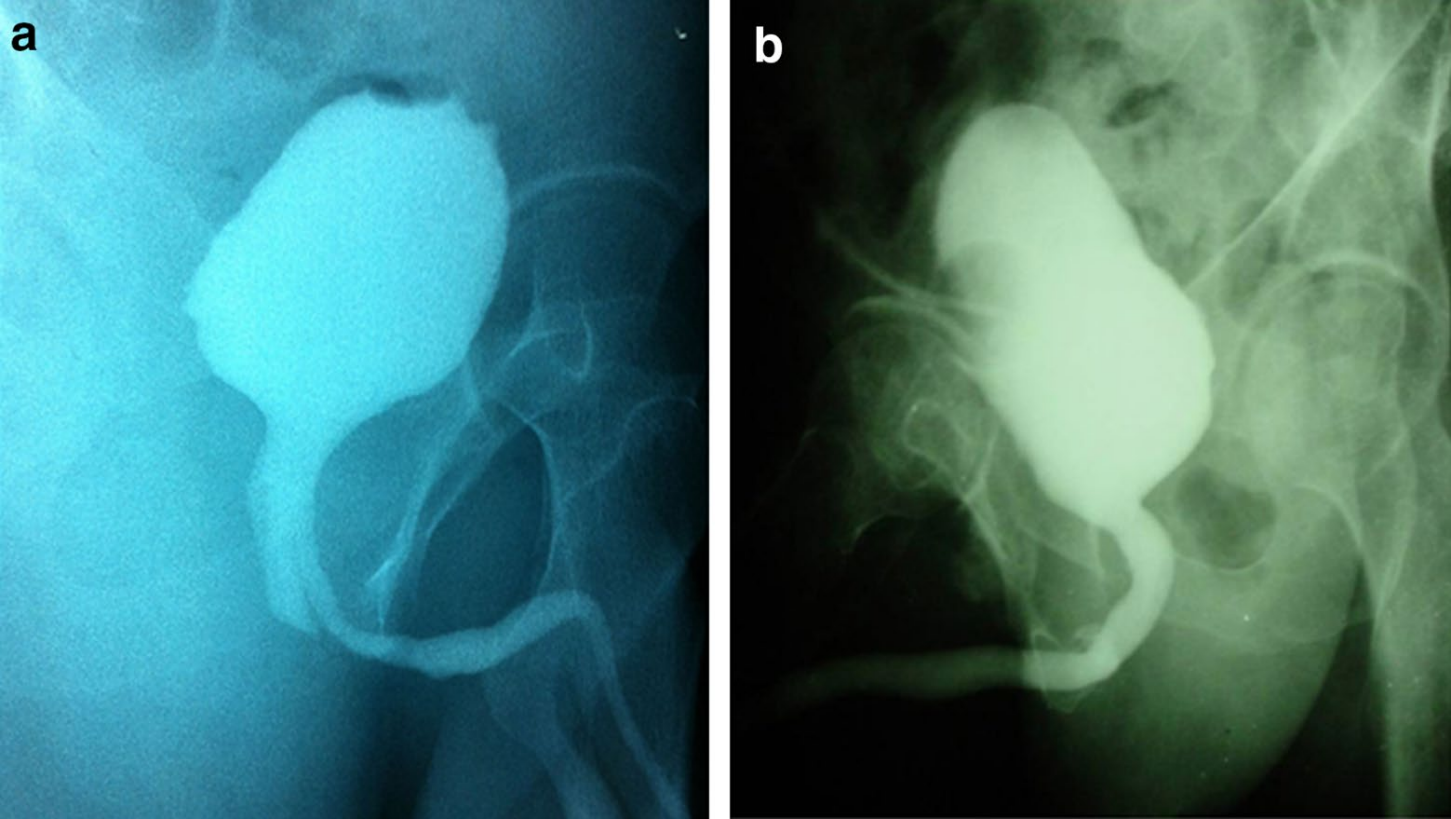
Urethrography. **a** Urethrography preoperatively shows the location and path of the false passage. The wider is the proximal posterior urethra with a blind-end, while the thinner one is the false passage. **b** Postoperative urethrography showed wide patent urethra without stricture in the minimally-invasive operation group

All the patients underwent urethrography (Fig. 1a) and flexible urethro-cystoscopy before the operation, to confirm the presence of false passage and identify their normal urethras through verumontanum (which is located in the normal urethra). Preoperative urethrography showed their stenotic urethral segments were <1.0 cm in length. Preoperative anti-infective therapy was given in each patient, and surgeries were conducted after a negative urine culture was obtained.

Open surgery procedures: an inverted Y-shaped incision in the perineum was made to dissociate the cavernous body of urethra to the stenotic segment. A flexible cystoscope was inserted through the bladder stoma, and a urethral dilator was inserted into the internal orifice of the normal urethra and advanced to the blind end of the proximal normal urethra with the assistance of the flexible cystoscope. The blind end of the normal urethra was incised from the perineal incision and the surrounding scar tissue was removed till the mucosa of the normal urethra was exposed. End-to-end anastomosis for the distal and proximal end of the urethra was conducted, and an F 18 Forley catheter was placed.

Endoscopic minimally-invasive procedures: an inflexible cystoscope was inserted from the bladder stoma. A urethral dilator was placed into the internal orifice of the normal urethra and advanced to the blind end of the proximal normal urethra with the assistance of the cystoscope. Then, a urethral resectoscope was inserted from the external urethral orifice to the narrow segment of the urethra, and located at the blind-end of the normal urethra under the guidance of the dilator in the proximal urethra. The blind-end of the normal urethra was incised with an internal-urethrotomy scalpel, and surrounding scars were removed with a resectoscope in case they were severe. False passage was excluded, zebra urological guide wire was placed into the normal urethra, and a F18 Forley catheter was inserted along the guide wire.

All the patients had their catheters removed 4 weeks after operation, and urination status was observed. Urethrography and urine flow rate examinations were performed 1 month after their catheter removal, and a re-examination of urine flow rate was done one year after the operation.

The postoperative urine flow rate and incontinence of the patients were statistically analyzed (t test) with the SPSS 18.0 software, and analysis results are considered statistically significant when p < 0.05. Of the two indicators, incontinence was evaluated with the *Questionnaire of International Consultant on Incontinence*.

### Ethics, consent and permissions

This study was approved by the Ethic Committee of Tongji Hospital Affiliated to Tongji University (2002-18-1J). This study was performed in accordance with the principles of Declaration of Helsinki. All subjects gave their consents to participate in this study.

### Consent to publish

Consent to publish has been obtained from the participants (or legal parent or guardian for children) to report individual patient data.

## Results

### Postoperative urethrography

In the open operation group, nine patients showed normal urination after catheters were removed after the operation, and urethrography also indicated unobstructed urethra, without any stenosis. Of these nine patients, one suffered from dysuresia after catheter removal, but got better after urethral dilatation.

In the minimally-invasive operation group, nine patients had normal urination without urethral stricture in urethrography (Fig. 1b), and two suffered from continuous dysuresia after the operation, which was improved after open operation treatment or urethral dilatation was performed respectively.

### Postoperative urine flow rate

**Fig. 2:**
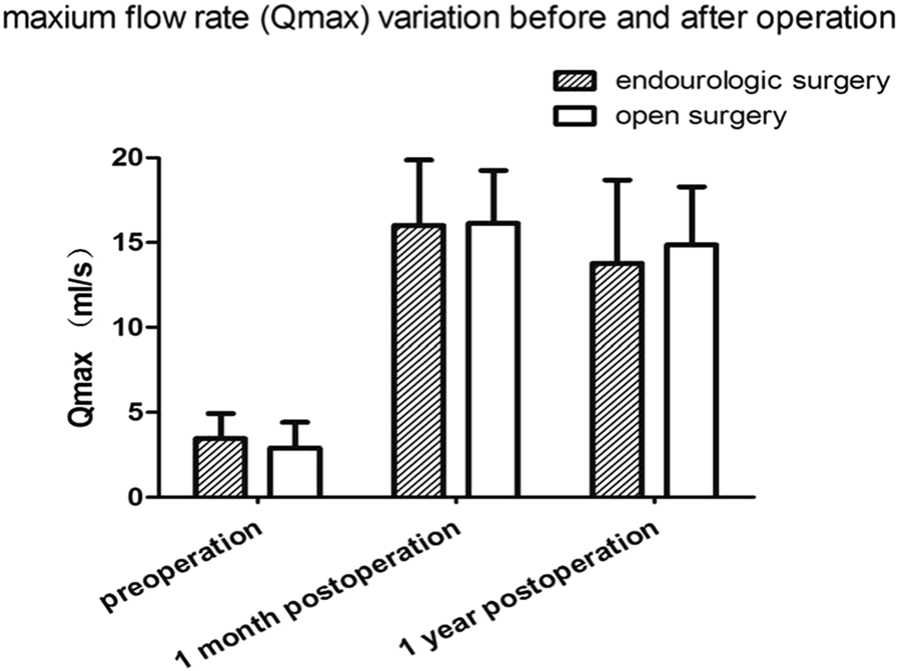
Maximum urinary flow rate. There is statistically significant improvement in post-operative urine flow rate for all the patients in both groups, p < 0.05

Changes in urine flow rates of the minimally-invasive operation group and the open operation group were obtained at the follow-ups conducted 1 month and one year after operation. Results showed that there was statistically significant improvement in post-operative urine flow rate of all the patients in both groups (p < 0.05), and the difference between the two groups was not statistically significant (p > 0.05, Table 1; Fig. 2).

**Table 1:** Post-operation urine flow rate

	Preoperative	Postoperative (1 month)	Postoperative (1 year)
Maximum urinary flow rate (ml/s)			
Minimally-invasive operation	3.45 ± 1.50	16.00 ± 3.88	13.77 ± 4.92
Open operation	2.90 ± 1.52	16.15 ± 3.10	14.85 ± 3.44

### Comparison of postoperative urinary incontinence

**Fig. 3:**
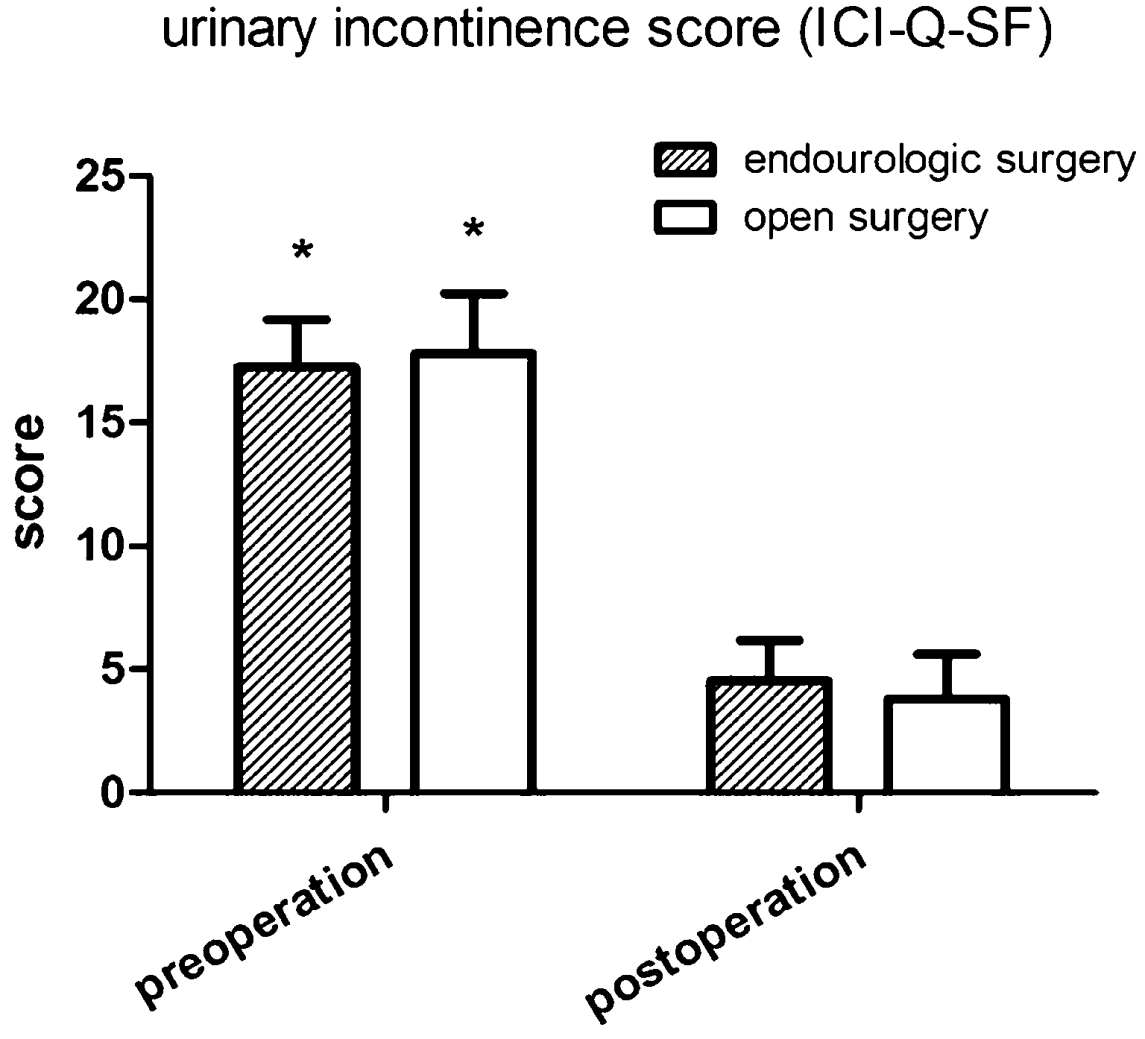
Comparison of urinary incontinence after surgery in the minimally-invasive operation group and the open operation group. Patients in both groups obtained significant improvement in urinary incontinence half a year after operation, p < 0.01

Changes in urine flow rates of the minimally-invasive operation group and the open operation group were obtained before the operations and at the follow-ups conducted half a year after operation. Results showed that patients in both groups obtained significant improvement in urinary incontinence half a year after operation (p < 0.01), and the difference between the two groups was not statistically significant (p > 0.05, Table 2; Fig. 3).

**Table 2:** Comparison of urinary incontinence after surgery in the minimally-invasive operation group and the open operation group

	Minimally-invasive operation	Open operation
Urinary incontinence score ICI-Q-SF		
Preoperative	17.27 ± 1.90	17.8 ± 2.44
Postoperative (6 months)	4.54 ± 1.63	3.80 ± 1.81

*ICI*-*Q*-*SF* Questionnaire of International Consultant on Incontinence

## Discussion

Most posterior-urethral false passages are secondary to urethral stricture caused by various reasons (Santucci et al. 2007), and is a pathological condition where the bladder and anterior urethra is connected by abnormal urethra, with iatrogenic urethral dilatation as its main contributor (Chiang and Dewan 2007). Most patients with false passage are also afflicted with dysuresia and urinary incontinence. During dilations, the operation is conducted from bypass into the bladder, and a false passage forms, thus causing urinary incontinence.

Preoperative urethrography and flexible cystoscopy are necessary for the diagnosis of post-traumatic urethral stricture with false passage (Li et al. 2014; Dogan et al. 2014). In the study, all patients received both prograde and retrograde urethrography and flexible cystoscopy before operation. Of the two examinations, urethrography was used to determine the presence of any abnormal passage in the urethra, and pre-operative flexible cystoscopy was helpful to detect possible false passage around the internal urethral orifice, and to distinguish a normal urethra from false passage, based on the presence of verumontanum structure in the passage.

Open operation is considered as a major means for treatment of posterior urethral stricture complicated with false passage, for its clear exposure, exact anastomosis, and fairly definite operational effect. However, open operation has some shortages, such as big operational wound, hemorrhage during operation, tendency of aggravated injury in surrounding tissues and neural, and especially the great influence on sexual functions. So, it is important to discover a treatment method with smaller trauma.

Intracavity minimally-invasive operation should be used with the presence of false passage and the distance being <1 cm, between the blind end of the proximal normal urethra and the distal urethra (i.e. length of stenotic passage), which has been confirmed by pre-operative urethrography and flexible cystoscopy. During the operation, a flexible cystoscope was inserted from the suprapubic bladder stoma, to find out the internal orifice of the urethra; and then, with the assistance of the cystoscope, a urethral dilator was placed at the internal orifice of the normal urethra and entered into the normal posterior urethra to prop up the blind end of the normal urethra; an internal-incision-scope was inserted from the external urethral orifice, to incise the blind end with cold-scalpel till the internal-incision-scope was able to enter into the bladder smoothly; the false passage was excluded; a zebra urological guide wire was inserted into the normal urethra, and a F 18 Forley catheter was placed along the wire.

Minimally-invasive operation should be conducted with the pre-condition that the distance between the blind end of the proximal normal urethra and the distal urethra (i.e. the length of the narrow segment) is <1.0 cm. A long stenotic segment brings difficulty to internal incision of the blind end, and also brings about poor localization as well as dissatisfying outcome. Therefore, open operation for patients with narrow segments of <1.0 cm is suggested.

False passage is not a normal anatomical passage, and the internal sphincter is unable to function when the external sphincter is damaged, so patients with false passage usually have urinary incontinence. The recovery of the normal anatomical structure of bladder-normal-urethra by surgery to restore the function of the internal urethral sphincter, is helpful in improving and recovering urinary incontinence (Podesta and Podesta 2015). In this study, incontinence was significantly improved in all patients, whether they received open operation or minimally-invasive operation. And there was no statistically significant difference in incontinence improvement between the two groups.

In the study, we collected data from postoperative urethrography, to evaluate the effects of two different methods in the treatment of urethral stricture complicated with false passage, and the data showed that there was no statistically significant difference between the outcome of minimally-invasive operation and open operation in treatment of urethral stricture complicated with false passage. Minimally-invasive operation was an alternative in addition to the open operation for the patients with posterior urinary stricture of <1.0 cm in length complicated with false passage.

However, minimally-invasive operation also has limitations: first, it is hard to perform a minimally-invasive operation on patients whose narrow segment of urethra is more than 1.0 cm in length, as complete removal of scars on narrow segment is out of reach in intracavitary treatment; second, it has poor effects on those patients with narrow urethral segment of <1.0 cm, who have had several urethral dilatations, resulting in severe urinary extravasation and solid scars. It has been exemplified by the two patients in our study, on whom the minimally-invasive operations failed, and both of them had received urethral dilatations more than 4 times; third, scar tissues on the narrow segment of urethra can not be completely excised in minimally-invasive operation in intracavitary treatment, but it could be in the open operation with end-to-end anastomosis of normal mucosa at two ends of urethra. Although no statistically significant difference was shown in the treatment effect in our study, studies with a larger sample size may have a different result (for example: the success rate of the minimally-invasive operation may be lower than that of the open operation).

In addition, no statistical analysis on sexual functions was performed on patients in the two groups. Theoretically, less local damage is caused in minimally-invasive operation, which is more helpful in protecting the sexual functions of patients. Further studies will continue to be conducted.

## Conclusions

Endoscopic minimally-invasive operation is an effective method for treatment of posterior urethral stricture with a fairly short narrow segment (<1.0 cm) and false passage. Compared with open operation, it has the advantages of smaller wound and quicker postoperative recovery, providing new treatment method in curing the urethral stricture complicated with false passage.
